# Zoonotic intestinal helminths interact with the canine immune system by modulating T cell responses and preventing dendritic cell maturation

**DOI:** 10.1038/s41598-017-10677-4

**Published:** 2017-09-04

**Authors:** Johannes Junginger, Katharina Raue, Karola Wolf, Elisabeth Janecek, Veronika M. Stein, Andrea Tipold, Anne-Rose Günzel-Apel, Christina Strube, Marion Hewicker-Trautwein

**Affiliations:** 1 Department of Pathology, University of Veterinary Medicine, Bünteweg 17, D-30559 Hannover, Germany; 2 Institute for Parasitology, Center for Infection Medicine, University of Veterinary Medicine, Bünteweg 17, D-30559 Hannover, Germany; 3 Unit of Reproductive Medicine of Clinics, University of Veterinary Medicine, Bünteweg 15, D-30559 Hannover, Germany; 4 Small Animal Clinic, University of Veterinary Medicine, Bünteweg 9, D-30559 Hannover, Germany; 5 Present Address: Vetsuisse Faculty, University of Bern, Länggassstrasse 128, CH-3012 Bern, Switzerland

## Abstract

Parasite co-evolution alongside the mammalian immune system gave rise to several modulatory strategies by which they prevent exaggerated pathology and facilitate a longer worm survival. As little is known about the immunoregulatory potential of the zoonotic canine parasites *Ancylostoma caninum* and *Toxocara canis* in the natural host, the present study aimed to investigate whether their larval excretory-secretory (ES) products can modulate the canine immune system. We demonstrated TcES to increase the frequency of CD4+ Foxp3^high^ T cells, while both AcES and TcES were associated with elevated Helios expression in Foxp3^high^ lymphocytes. ES products were further capable of inducing IL-10 production by lymphocytes, which was mainly attributed to CD8+ T cells. ES treatment of PBMCs prior to mitogen stimulation inhibited polyclonal proliferation of CD4+ and CD8+ T cells. Moreover, monocyte-derived ES-pulsed dendritic cells reduced upregulation of MHC-II and CD80 in response to lipopolysaccharide. The data showed that regulation of the canine immune system by *A. caninum* and *T. canis* larvae comprises the modification of antigen-specific and polyclonal T cell responses and dendritic cell maturation.

## Introduction

Parasites are responsible for public health problems worldwide with the highest prevalence being attributed to intestinal helminths^1–3^. Helminth infections occur in animals as well as humans^3, 4^, with *Ancylostoma caninum* and *Toxocara canis* being two of the most important intestinal nematodes in dogs. For instance, the prevalence of *T. canis* in the definitive host has been reported to range between 1.4 and 30.5% in Europe with the highest prevalence usually found in puppies^5, 6^. Although dogs are required for completion of the life-cycle, parasite larval stages are further capable of inducing diseases in paratenic hosts including humans. For example, *A. caninum*, which is closely related to the main human hookworms *A. duodenale* and *Necator americanus*, is responsible for cutaneous larva migrans and eosinophilic enteritis associated with abdominal pain^7, 8^. In contrast, *T. canis* larvae, the cause of human toxocariasis along with *T. cati*, migrate to various organs and thereby cause neurological, ocular or other systemic diseases in human patients^9, 10^.

Remarkably, during their co-evolution alongside the mammalian immune system, parasites have adopted several immunomodulatory mechanisms allowing them to prevent exaggerated pathology and thereby to facilitate a chronic, long-lasting course of infection enabling completion of their life-cycle^11–13^. The wide spectrum of immune modulation includes interaction with antigen-presenting cells, suppression of pro-inflammatory and induction of anti-inflammatory cytokines, as well as generation of regulatory T cells (Tregs)^14–17^. Related downstream effects are not only beneficial for the worm itself, but being in a parasite-induced pro-regulatory condition can have further bystander consequences for the host^18^. For instance, parasitic infections have been shown to suppress chronic inflammatory disorders such as inflammatory bowel disease^19^. Interestingly, this raises the question as to whether parasite-related mechanisms represent suitable targets for novel therapeutic strategies^20–22^. Remarkably, it has also been demonstrated that parasite-related immune modulation can have further effects including a reduced response to other pathogens or vaccines^23–25^.

Several studies have shown larval and adult parasitic secretions to play a central role in the parasite-host interaction^26–28^. These so-called ‘excretory-secretory’ (ES) products are mainly released from the parasite’s cuticle, oral cavity and secretory glands, and therefore represent the direct interface between the parasite and the host^29^. As revealed by proteomic studies, they comprise a complex mixture of enzymes, mucins, lectins, proteoglycans, other proteins, carbohydrates and lipids^29–31^. ES products have also been shown to directly interact with the host’s immune system^32^. For example, this can include the induction of the Treg-specific transcription factor Foxp3 as known for *Heligmosomoides polygyrus* infection in mice^16^, or binding the CD11b/CD18 integrin on neutrophils by glycoproteins leading to reduced hydrogen peroxide production as demonstrated for *Haemonchus contortus* ^33^.

Most of the evidence concerning how *A. caninum* and *T. canis* interact with the immune system is based on murine and human studies. For instance, infection with adult *A. caninum* is capable of suppressing intestinal pathology in dextran sodium sulphate-induced murine colitis, which is associated with dominance of Th2-related but suppression of pro-inflammatory cytokines, and recruitment of alternatively activated macrophages and eosinophils^34^. Similar effects were observed when human peripheral blood mononuclear cells (PBMCs) were stimulated with larval/adult ES antigens (AcES) or co-cultivated with living third stage (L3) larvae^17^. Interestingly, the suppression of pro-inflammatory cytokines and the anti-proliferating effects of AcES are significantly increased in patients infected with *A. caninum* compared to uninfected controls^17^. Enhancing immunosuppression by inducing anti-inflammatory cytokines, such as TGF-β and/or IL-10, has also been shown for *T. canis* in humans and mice^14, 35, 36^. Its anti-inflammatory potential is further evidenced by *in vivo* data demonstrating Foxp3 mRNA and protein levels to be increased in *T. canis*-infected mice^37^. In addition, murine cytokine responses in the context of *T. canis* infection are further predominated by high levels of Th2-related cytokines such as IL-4 and IL-5^35, 38^.

In contrast to studies investigating the immune response in humans and mice, little is known about the interaction of *A. caninum* and *T. canis* with the canine immune system. While investigations into the immunomodulatory effects of *A. caninum* in the natural host are lacking so far, a few studies suggest an anti-inflammatory potential of *T. canis* in dogs. For instance, increased IL-10 production was demonstrated for PBMCs of pregnant dogs when re-exposed to larval *T. canis* ES (TcES) *in vitro*, this being associated with lower production of IFN-γ^39^. More recently, a low IFN-γ/IL-10 ratio and dominance of IL-5 was found when canine PBMCs were stimulated with extracts of adult *T. canis* ^40^. Besides investigations focussing on cytokine responses, we recently demonstrated that Foxp3+ Tregs are elevated in the intestinal mucosa of nematode-infected animals, suggesting that parasite-related immune modulation in dogs may also occur on a cellular level^41^. As responsible mechanisms are still unknown, the aim of the present study was to further elucidate the anti-inflammatory effects of *A. caninum* and *T. canis* larval ES products on canine T cells and dendritic cells (DCs) *in vitro*.

## Results

### TcES increases Foxp3^high^ expression in canine CD4+, CD4+ CD8+ and CD4− CD8− T cells

**Figure 1:**
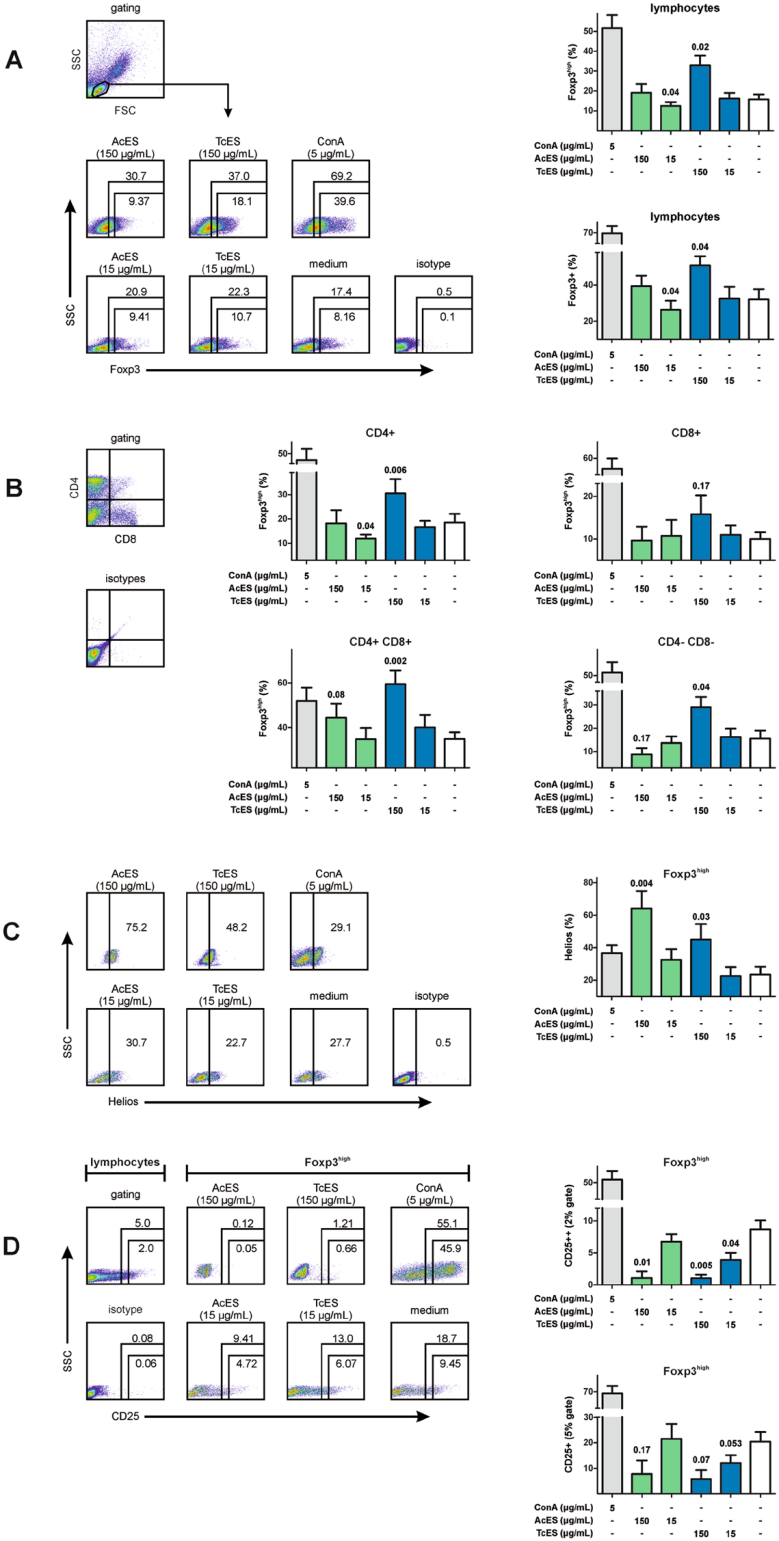
Tregs inducing assay. (**A**) Treatment of canine PBMCs with TcES at 150 µg/mL caused an increase in Foxp3+ and Foxp3^high^. Although single individuals show increased frequencies of Foxp3+ lymphocytes after treatment with AcES at 150 µg/mL (illustrated by the flow cytometric plots) this effect did not achieve statistical significance. In contrast, AcES at 15 µg/mL was associated with a slight decrease in Foxp3 expression in lymphocytes. (**B**) Three-colour flow cytometry revealed the TcES-associated increase in Foxp3^high^ lymphocytes to be associated with CD4+, CD4+ CD8+ double-positive and CD4- CD8- double-negative subsets, while this effect was lower in CD8+ T cells. Compared to TcES, treatment with AcES at 150 µg/mL was associated with a much lower elevation in Foxp3^high^ expression by lymphocytes and CD4+ CD8+ T cells. (**C**) Cultivating the cells in the presence of 150 µg/mL AcES or TcES induced marked increase in Helios expression by Foxp3^high^ lymphocytes. (**D**) Treatment of canine PBMCs with ES products was associated with decreased frequencies of CD25+ and CD25++ cells within Foxp3^high^ lymphocytes and this effect was most obvious at 150 µg/mL. P-values were calculated according to paired t-test. Error bars represent standard error of mean (SEM). FSC = forward scatter, SSC = side scatter. Experiments were repeated six times at two different time points.

For estimating the immunoregulatory capacities of *A. caninum* and *T. canis* in the definitive host, larval ES products were used to stimulate canine immune cells *in vitro*. To evaluate whether *A. caninum* and *T. canis* larvae share a Treg inducing ability, canine PBMCs were cultured in the presence of AcES and TcES for 72 hours and then analysed with respect to their Foxp3 expression by flow cytometry. This approach demonstrated TcES to be capable of increasing the frequency of Foxp3^high^ and Foxp3+ lymphocytes at 150 µg/mL (means of Foxp3^high^ cells: medium 15.8%, 150 µg/mL TcES 32.9% with p = 0.02; means of Foxp3+ lymphocytes: medium 32.1%, 150 µg/mL TcES 50.8% with p = 0.04; Fig. 1A). Co-staining for CD4 and CD8 revealed the TcES-associated increase in Foxp3^high^ expression to be associated with CD4+, CD4 + CD8+ and CD4- CD8- double-negative T cells (means of Foxp3^high^ in CD4+ T cells: medium 18.5%, 150 µg/mL TcES 30.5% with p = 0.006; means of Foxp3^high^ in CD4+ CD8+ cells: medium 34.9%, 150 µg/mL TcES 59.5% with p = 0.002; means of Foxp3^high^ in CD4- CD8- cells: medium 15.6%, 150 µg/mL TcES 29.0% with p = 0.04; Fig. 1B). Treatment with AcES at 150 µg/mL was associated with a much lower increase in frequencies of Foxp3^high^ cells within lymphocytes as well as CD4 + CD8+ T cells compared to TcES (means of Foxp3^high^ in lymphocytes: 150 µg/mL AcES 19.0% with p = 0.58; means of Foxp3^high^ in CD4 + CD8+ T cells: 150 µg/mL AcES 44.5%, p = 0.08; Fig. 1B). In contrast, AcES at 15 µg/mL was associated with a slight decrease in Foxp3^high^ cells in lymphocytes and CD4+ T cells (means of Foxp3^high^ in lymphocytes: 15 µg/mL AcES 12.4% with p = 0.04; means of Foxp3^high^ in CD4+ T cells: 15 µg/mL AcES 12.0%, p = 0.04; Fig. 1A and B).

### AcES and TcES elevate Helios expression in canine Foxp3^high^ lymphocytes

Incubation of PBMCs in the presence of AcES as well as TcES at 150 µg/mL caused Foxp3^high^ lymphocytes to significantly increase the expression of Helios (means: medium 23.6%, AcES 64.1% with p = 0.004, TcES 45.1% with p = 0.03; Fig. 1C). This effect was much lower for AcES at 15 µg/mL, while it was absent after treatment with TcES at 15 µg/mL (means: 15 µg/mL AcES 32.5% with p = 0.13, 15 µg/mL TcES 22.6% with p = 0.71).

### AcES and TcES decrease CD25++ Foxp3^high^ lymphocytes

Analysing the co-expression of Foxp3 and CD25 showed ES products to decrease CD25++ Foxp3^high^ lymphocytes at 150 µg/mL (means: 150 µg/mL AcES 1.1% with p = 0.01, 150 µg/mL TcES 1.1% with p = 0.005, medium 8.7%; Fig. 1D). The decline in CD25++ Foxp3^high^ lymphocytes was much lower at 15 µg/mL (means: 15 µg/mL AcES 6.8% with p = 0.32, 15 µg/mL TcES 3.9% with p = 0.04). This effect was less distinct with loss of statistical significance when analysing the CD25+ subset in Foxp3^high^ lymphocytes (means: 150 µg/mL AcES 7.7% with p = 0.17, 15 µg/mL AcES 21.5% with p = 0.87, 150 µg/mL TcES 5.7% with p = 0.07, 15 µg/mL TcES 12.0% with p = 0.053, medium 20.4%).

### AcES and TcES induce IL-10 production by canine lymphocytes

**Figure 2:**
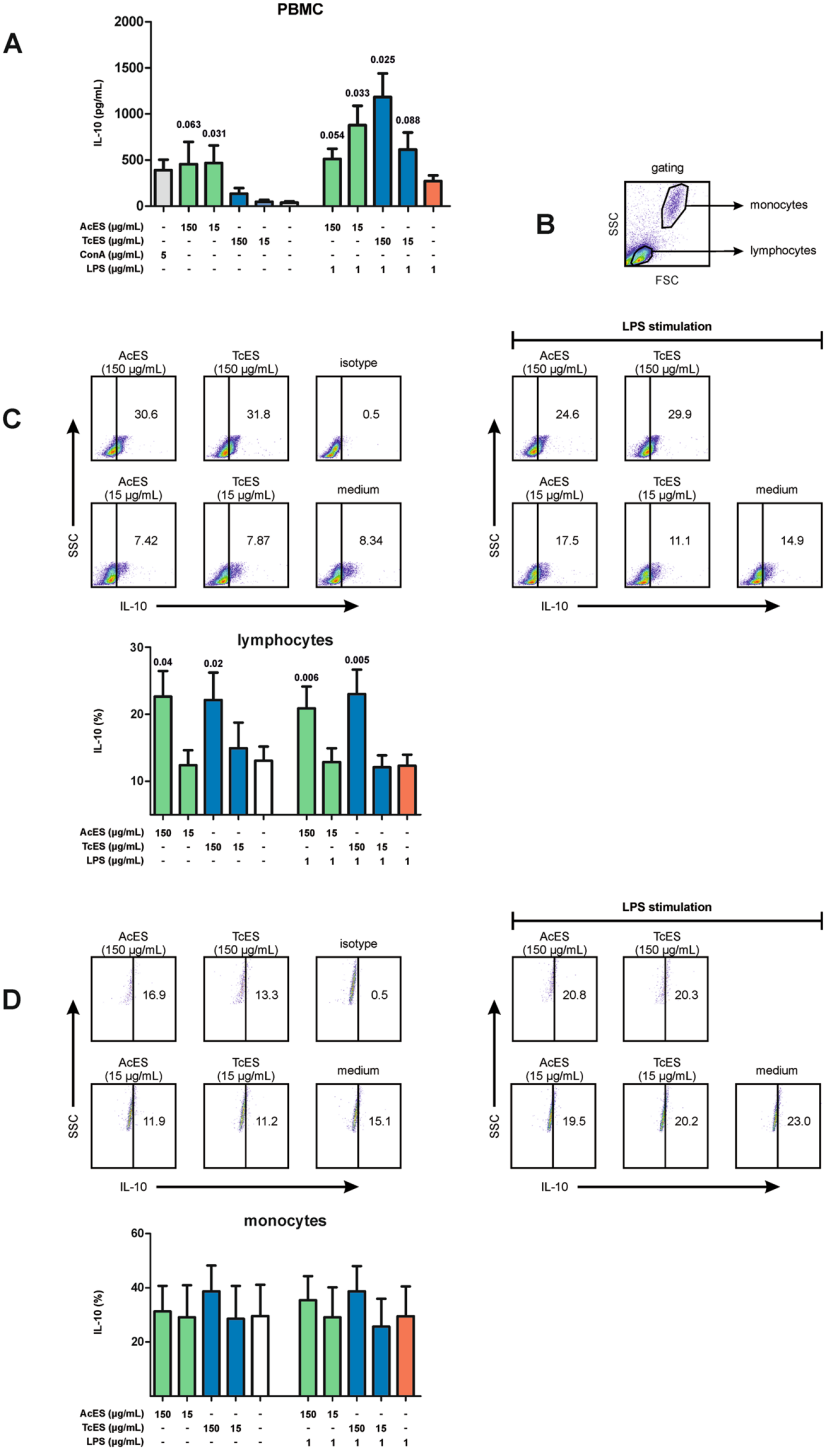
IL-10 expression. (**A**) ELISA revealed both AcES and TcES to be capable of increasing IL-10 secretion by canine PBMCs. For AcES this effect was more obvious at 15 µg/mL. In contrast, TcES-associated IL-10 secretion was stronger at 150 µg/mL and was only detected when cells were co-stimulated with LPS. (**B**) For analysis of intracellular IL-10 staining, PBMCs were gated on lymphocytes and monocytes based on side (SSC) and forward scatter (FSC) morphology. This revealed (**C**) lymphocytes but not (**D**) monocytes to be responsible for ES-induced IL-10 expression and the effect was independent from stimulation with LPS. P-values were calculated according to Wilcoxon signed rank test (ELISA without LPS stimulation) or paired t-test (ELISA with LPS stimulation, intracellular cytokine staining). Error bars show standard error of mean (SEM). Experiments were repeated six times at two different time points.

For investigating whether ES antigens are capable of increasing IL-10 production, PBMCs were pulsed with AcES or TcES following stimulation with lipopolysaccharide (LPS) or medium. Analysis of culture supernatants using a canine IL-10 sandwich enzyme-linked immunosorbent assay (ELISA) showed both AcES and TcES to be capable of increasing IL-10 secretion (Fig. 2A). For AcES this effect was higher at 15 µg/mL, while TcES-associated IL-10 secretion was stronger at 150 µg/mL and was only present when cells were co-stimulated with LPS (means of IL-10 in pg/mL: 15 µg/mL AcES: 468.1, p = 0.031; 150 µg/mL AcES: 455.7, p = 0.063; 15 µg/mL AcES/LPS: 878.7, p = 0.033; 150 µg/mL AcES/LPS: 512.7, p = 0.054; 15 µg/mL TcES: 47.5, p = 0.44; 150 µg/mL TcES: 133.6, p = 0.22; 15 µg/mL TcES/LPS: 613.8, p = 0.088; 150 µg/mL TcES/LPS: 1184.6, p = 0.025; medium: 37.5; LPS: 271.0). To verify changes in IL-10 expression on the single cell level, intracellular cytokine staining was performed (Fig. 2B-D). This confirmed ES products to slightly increase IL-10 expression at 150 µg/mL and further showed that lymphocytes but not monocytes are responsible for this effect (means of IL-10+ in lymphocytes: medium: 13.1%; LPS: 12.3%; 150 µg/mL AcES: 22.6%, p = 0.04; 150 µg/mL AcES/LPS: 20.9%, p = 0.006; 150 µg/mL TcES: 22.1%, p = 0.02; 150 µg/mL TcES/LPS: 23.0%, p = 0.005; 15 µg/mL AcES: 12.4%; 15 µg/mL AcES/LPS: 12.9%; 15 µg/mL TcES: 14.9%; 15 µg/mL TcES/LPS: 12.1%).

### ES-induced IL-10 expression is mainly related to CD8+ T cells

**Figure 3:**
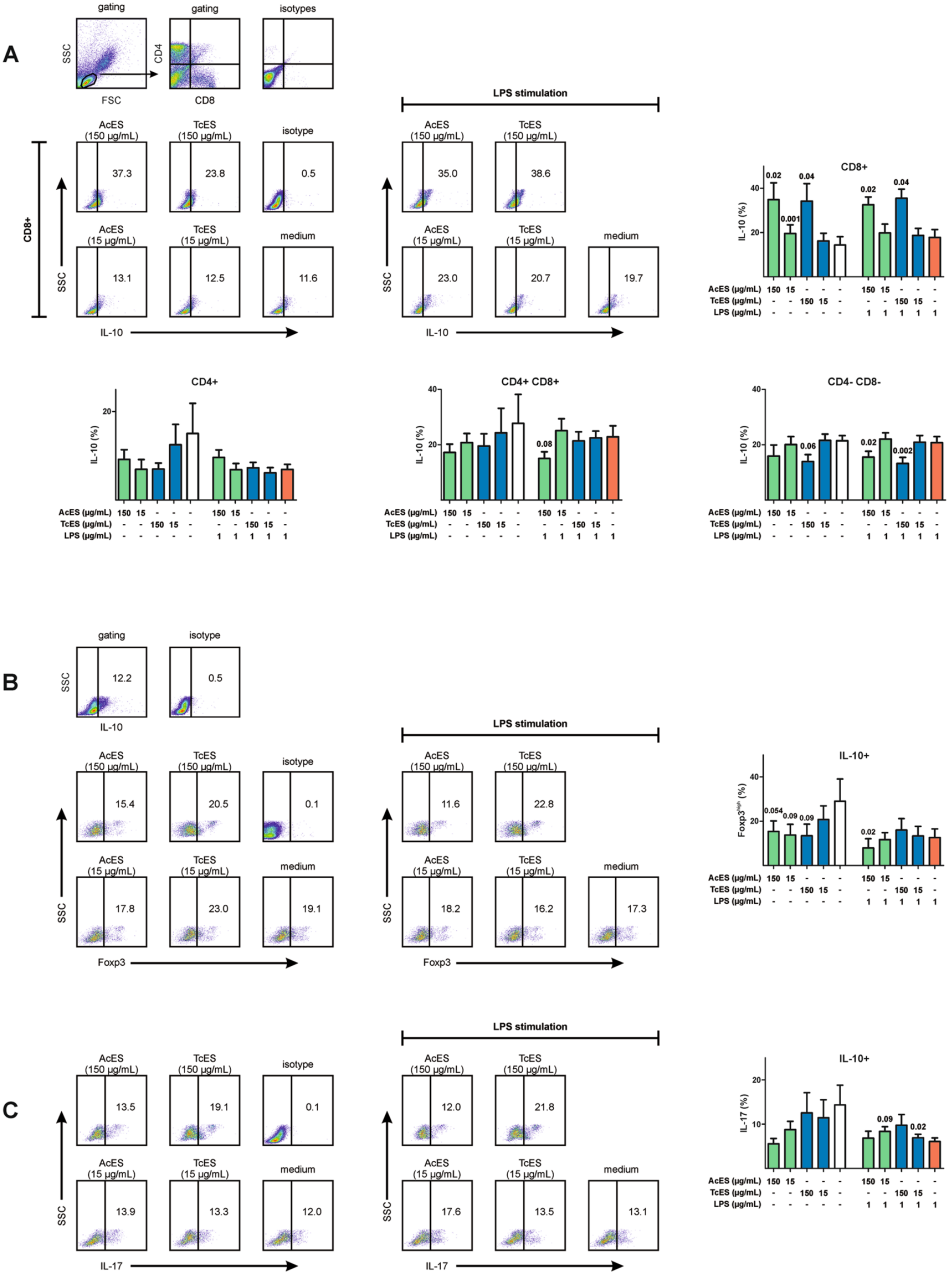
Characterisation of IL-10+ lymphocytes. (**A**) Three-colour flow cytometry showed CD8+ lymphocytes to be the main source of ES-induced IL-10 expression. In contrast, CD4- CD8- double-negative lymphocytes revealed lower IL-10 expression after ES treatment at 150 µg/mL. (**B**) ES antigens were associated with a mild decrease in Foxp3^high^ cells within IL-10+ lymphocytes in the absence of LPS stimulation, although the finding failed to reach statistical significance. For AcES at 150 µg/mL, this effect was more obvious when cells were stimulated with LPS (**C**) Co-staining for IL-17 showed ES antigens to be capable of elevating IL-17 expression in LPS-stimulated IL-10+ lymphocytes and this effect was significant for TcES at 15 µg/mL. Although the data may suggest a decrease in IL-17 expression by unstimulated IL-10+ lymphocytes this effect failed to reach statistical significance. P-values were calculated according to paired t-test. Error bars illustrate standard error of mean (SEM). FSC = forward scatter, SSC = side scatter. Experiments were repeated six times at two different time points.

To further characterise the phenotype of IL-10+ lymphocytes, we used three-colour flow cytometry for IL-10, CD4 and CD8 (Fig. 3A). Gating on different T cell subsets revealed CD8+ T cells as being the main source of ES-induced IL-10 (means of IL-10+ in CD8+ T cells: medium: 13.9%; LPS: 16.1%; 150 µg/mL AcES: 26.6%, p = 0.02; 150 µg/mL AcES/LPS: 25.2%, p = 0.02; 150 µg/mL TcES: 26.2%, p = 0.04; 150 µg/mL TcES/LPS: 27.0%, p = 0.04; 15 µg/mL AcES: 17.1%, p = 0.001; 15 µg/mL AcES/LPS: 13.3%; 15 µg/mL TcES: 15.1%; 15 µg/mL TcES/LPS: 16.6%).

In contrast, treatment with ES at 150 µg/mL caused a mild decrease in IL-10 within the CD4- CD8- double-negative subset and this effect was more obvious when cells were stimulated with LPS (medium: 19.8%; LPS: 19.1%; 150 µg/mL AcES: 14.6%, p = 0.3; 150 µg/mL AcES/LPS: 14.3%, p = 0.02; 150 µg/mL TcES: 12.8%, p = 0.06; 150 µg/mL TcES/LPS: 12.2%, p = 0.002; 15 µg/mL AcES: 18.5%; 15 µg/mL AcES/LPS: 20.4%; 15 µg/mL TcES: 19.9%; 15 µg/mL TcES/LPS: 19.3%).

Analysis of Foxp3 expression in IL-10+ lymphocytes showed varying amounts of IL-10+ Foxp3^high^ cells ranging from 0.2 up to 68.4% (mean: 19.1%; Fig. 3B). Although the data suggest ES products to slightly diminish the frequencies of IL-10+ Foxp3+ cells in the absence of LPS stimulation, this effect did to gain statistical significance. For AcES at 150 µg/mL, however, this effect was more obvious in the presence of LPS (LPS: 15.6%; 150 µg/mL AcES/LPS: 9.8%, p = 0.02).

Co-staining for IL-10 and IL-17 showed low frequencies of IL-10+ lymphocytes to express IL-17 (mean: 18.7%; Fig. 3C) although co-expression was higher (up to 67.6%) in single individuals. Interestingly, ES antigens were capable of elevating IL-17 expression in LPS-stimulated IL-10+ lymphocytes which gained statistical significance for TcES at 15 µg/mL (means of IL-17+ in LPS-stimulated IL-10+ lymphocytes: LPS: 12.6%; 150 µg/mL AcES: 14.1%, p = 0.53; 15 µg/mL AcES: 17.3%, p = 0.09; 150 µg/mL TcES: 20.1%, p = 0.17; 15 µg/mL TcES: 14.3%, p = 0.02). Although the data may further suggest a decrease in IL-17 expression by unstimulated IL-10+ lymphocytes this trend was not significant.

### AcES and TcES inhibit polyclonal T cell proliferation

**Figure 4:**
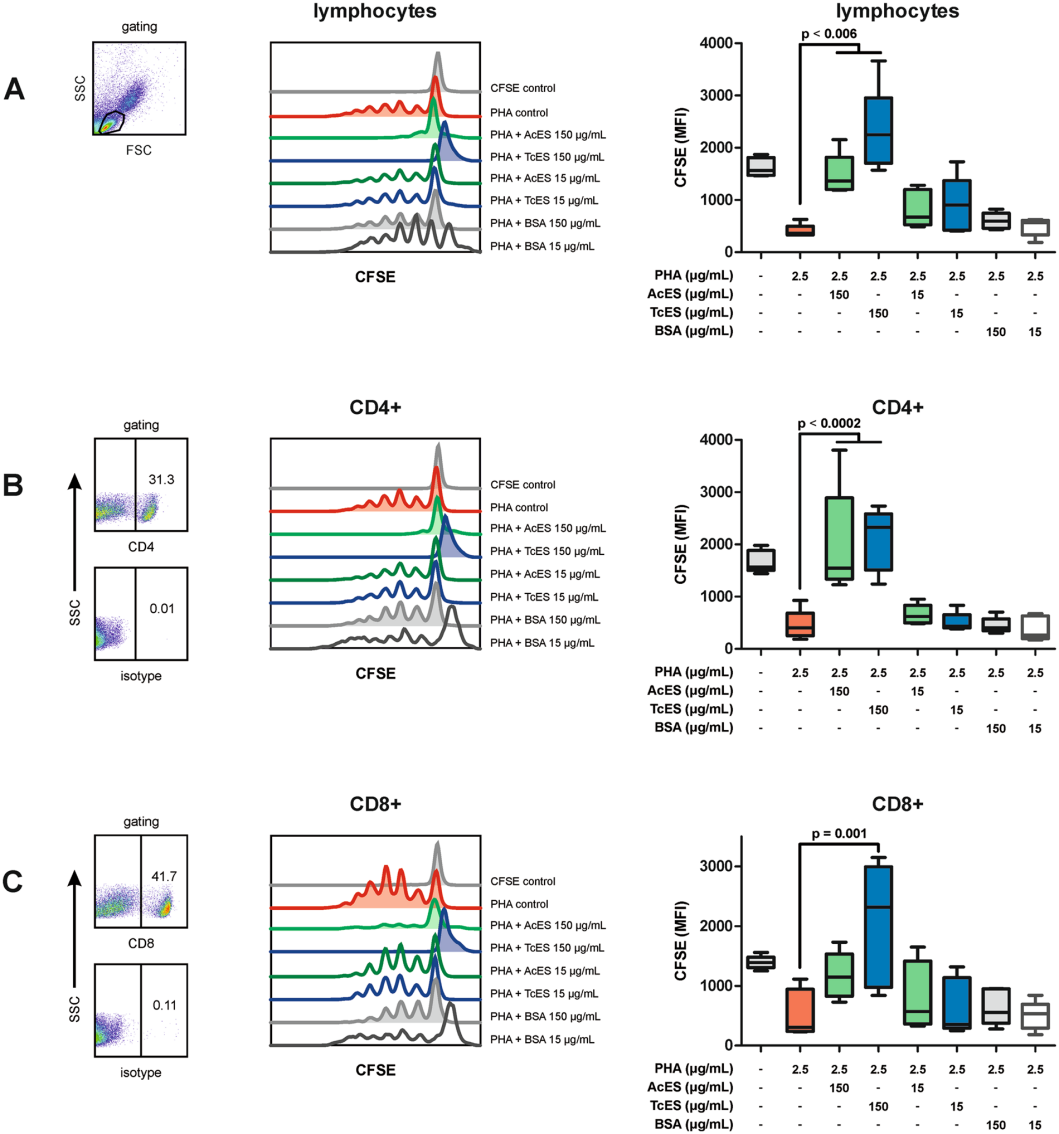
T cell proliferation assay. Cultivating CFSE-stained PBMCs in the presence of ES antigens revealed TcES to be capable of reducing PHA-induced lymphocyte proliferation (**A**) at 150 µg/mL and this was detectable in both CD4+ (**B**) and CD8+ (**C**) T cells. AcES showed similar effects in lymphocytes and CD4+ cells, while the anti-proliferative capability was lower with loss of statistical significance in CD8+ T cells. P-values were calculated according to one-way ANOVA followed by the Tukey’s honest significant difference post-hoc test. Error bars represent standard error of mean (SEM). FSC = forward scatter, SSC = side scatter. Experiments were repeated six times at two different time points.

To evaluate whether ES antigens are capable of reducing polyclonal T cell proliferation, carboxyfluorescein succinimidyl ester (CFSE)-labelled PBMCs were stimulated with phytohaemagglutinin-L (PHA-L) for 4 days and proliferation of lymphocytes as well as CD4 and CD8 subsets was then analysed using flow cytometry. This demonstrated pulsing with TcES at 150 µg/mL to significantly prevent mitogen-induced proliferation of lymphocytes (Fig. 4A) and this effect was present in both CD4+ (Fig. 4B) and CD8+ (Fig. 4C) cells (CFSE mean fluorescence intensity, MFI, of lymphocytes: 150 µg/mL TcES 2313.6 with p < 0.001, PHA-L control 404.4; CD4+ T cells: 150 µg/mL TcES 2104.4 with p < 0.001, PHA-L control 454.4; CD8+ T cells: 150 µg/mL TcES 2051 with p = 0.001, PHA-L control 533.6). AcES revealed a similar effect on the proliferation of lymphocytes and CD4+ T cells, while the anti-proliferative capability was lower with loss of statistical significance in CD8+ T cells (CFSE MFI for lymphocytes: 150 µg/mL AcES 1480.8 with p = 0.006; CD4+ T cells: 150 µg/mL AcES 1999.6 with p = 0.0002; CD8+ T cells: 150 µg/mL AcES 1173.8 with p = 0.5). To rule out unspecific effects of proteins, such as covering of mitogen receptors, being responsible for this observation, ES products were replaced by bovine serum albumin (BSA) at 15 and 150 µg/mL as a further control. However, in contrast to ES products, BSA did not reduce PHA-L-induced proliferation of lymphocytes, CD4+ or CD8+ T cells (CFSE MFI lymphocytes: 150 µg/mL BSA 601.8 with p = 0.99, 15 µg/mL BSA 489.8 with p = 0.99; CD4+ T cells: 150 µg/mL BSA 449.4 with p = 1.0, 15 µg/mL BSA 385.12 with p = 0.99; CD8+ T cells: 150 µg/mL BSA 639.8 with p = 0.99, 15 µg/mL BSA 500.02 with p = 1.0).

### AcES and TcES impair LPS-induced maturation of canine moDCs

**Figure 5:**
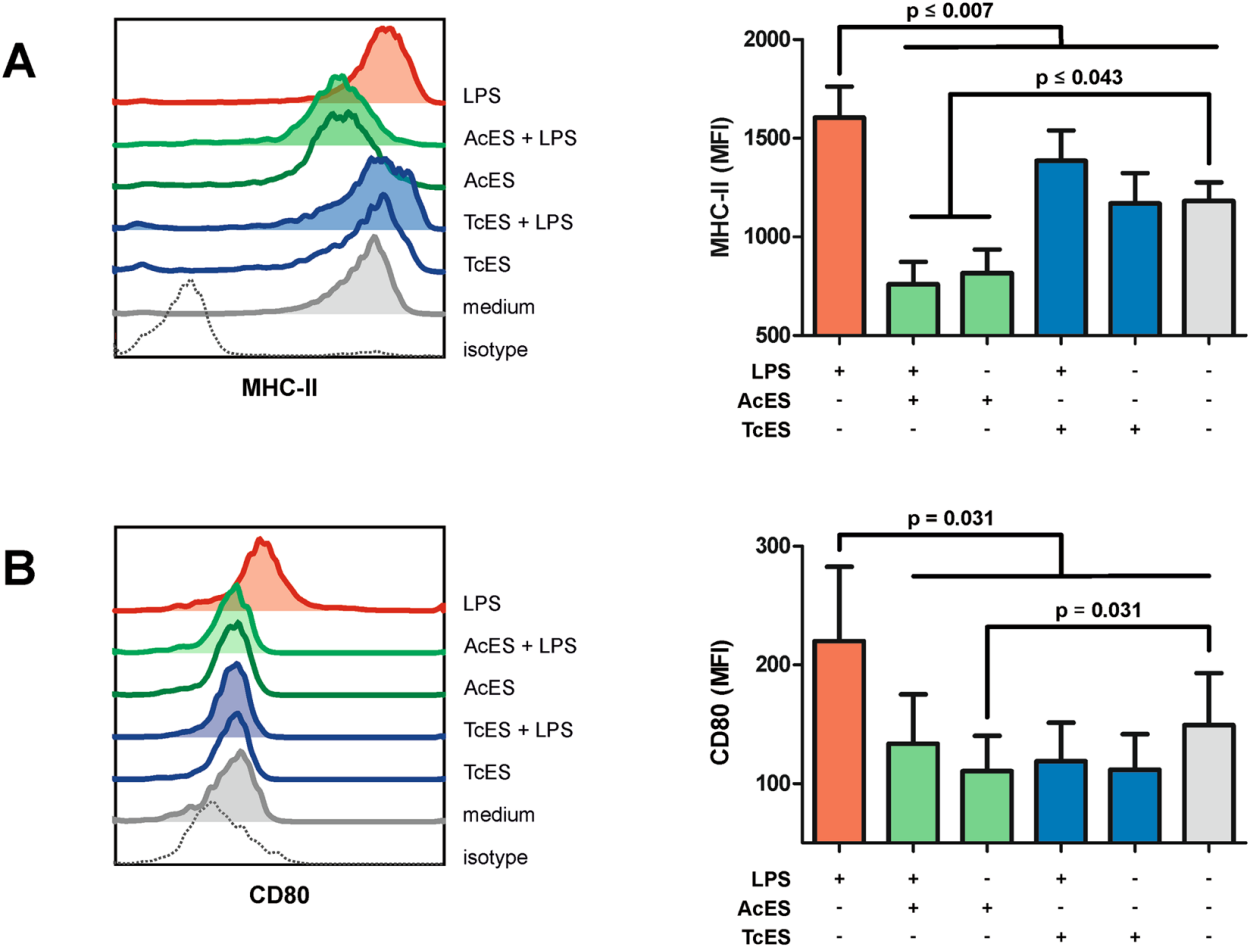
DC maturation assay. Pulsing of moDCs with ES products before LPS stimulation revealed both AcES and TcES to be capable of preventing the upregulation of MHC-II (**A**) and CD80 (**B**). For MHC-II, AcES was exceeding the effect of TcES by causing moDCs to downregulate MHC-II expression compared to medium control. Although ES preparations caused downregulation of CD80 compared to medium control this effect was statistically significant only for AcES without subsequent LPS stimulation. P-values were calculated according to paired t-test (for MHC-II) and Wilcoxon signed rank test (for CD80). Error bars show standard error of mean (SEM). Experiments were repeated six times at two different time points.

To investigate the effect of ES antigens on the maturation of monocyte-derived DCs (moDCs), flow cytometry was used to analyse their expression of major histocompatibility complex class-II (MHC-II) and the co-stimulatory molecule CD80 after Toll-like receptor 4 (TLR4) stimulation. This demonstrated pulsing of moDCs with TcES before LPS stimulation to significantly reduce their MHC-II upregulation (MFI: TcES/LPS 1386.03 with p = 0.001, TcES 1169.23 with p = 0.0009, medium control 1182.43 with p = 0.005, LPS control 1603.85; Fig. 5A). Interestingly, excelling the effect of TcES, pulsing of moDCs with AcES resulted in MHC-II expression lower than that of the medium control and this effect was independent from the presence of LPS (MFI: AcES/LPS 759.68 with p = 0.03, AcES 816.08 with p = 0.04). This was in contrast to pulsing with TcES without subsequent LPS stimulation which did not change the baseline MHC-II expression of moDCs (MFI: 1169.23 with p = 0.9).

Similar to MHC-II, LPS-induced upregulation of CD80 was prevented when moDCs were pulsed with ES preparations (MFI: AcES/LPS 133.68 with p = 0.031, AcES 110.5 with p = 0.031, TcES/LPS 118.84 with p = 0.031, TcES 111.68 with p = 0.031, LPS control 220.1, medium control 149.18 with p = 0.031). In addition, expression of CD80 by ES-pulsed moDCs was lower than for medium control and this effect achieved statistical significance for AcES without subsequent LPS stimulation (p = 0.031).

## Discussion

Modulating and suppressing the immune system is an important skill of many parasites by which they are able to influence the host’s immune response^26^. However, little is known about the immunomodulatory pathways adopted by canine parasites during their co-evolution alongside the dog’s immune system. We recently demonstrated that Foxp3+ lymphocytes are elevated in the intestinal mucosa of nematode-infected dogs *in vivo* and hypothesised a Treg-inducing potential for canine intestinal helminths in the natural host^41^. Therefore the present study aimed to investigate whether the zoonotic organisms *A. caninum* and *T. canis* mediate immunosuppression in dogs and whether this includes the induction of Tregs. Confirming our hypothesis, the results demonstrated CD4+ Foxp3^high^ T cells to be elevated in PBMCs after cultivation in the presence of TcES. This finding is in accordance with observations in mice demonstrating Foxp3 to be increased on both the mRNA and protein levels in *T. canis*-infected animals and interestingly this effect was enhanced when mice obtained TcES prior to infection^37^. Additionally, a Treg-inducing ability is also known for several murine helminths including *Brugia malayi* ^42^, *H. polygyrus* ^43^ and *Strongyloides ratti* ^44^. Tregs comprise a small Th subset with anti-inflammatory activity by which the immune system can maintain a homeostatic environment in the steady state and prevent exaggerated inflammation during immune responses on a cellular level^45, 46^. This is of particularly high importance in the context of the intestinal mucosa as induction and expansion of Tregs is essential for establishing oral tolerance^46^. Considering the immunoregulatory potential of intestinal parasites, several studies hypothesised their therapeutic potential for chronic inflammatory disorders such as inflammatory bowel disease, which is hallmarked by a breakdown of intestinal homeostasis associated with a decline in mucosal Tregs^19, 20, 41^. Interestingly, TcES also caused increased Foxp3 expression in CD4+ CD8+ double-positive T cells. In dogs, this subset represents a heterogeneous population of activated T cells in the peripheral blood that can develop from CD4+ and CD8+ T cells^47^. Our observation that these cells express Foxp3 is in accordance to a previous study on canine Tregs in the peripheral blood and popliteal lymph node^48^. Although canine CD4+ CD8+ T cells are suggested to exert a regulatory function^49^, the targets of this mechanism still remain unknown. Therefore, additional studies are needed to further investigate the role of canine CD4+ CD8+ double-positive T cells in helminth infection.

Treatment with ES extracts was not associated with elevation of the CD25++ compartment of Foxp3^high^ lymphocytes which was in contrast to the effect of Concanavalin A (ConA). CD25 is classically linked to the phenotype of canine Tregs and the degree of CD25 expression in ConA-stimulated CD4+ T cells is correlated with Treg function suggesting CD25++ Foxp3^high^ T cells to be activated canine Tregs^48^. The results may therefore suggest ES products to induce canine Tregs in a less activated state. However, this finding may otherwise indicate that the phenotype of activated canine Tregs can differ depending on the stimulus (e.g., ConA versus parasitic antigen), which needs to be investigated in further studies.

Compared to TcES, treatment with AcES caused a lower increase in Foxp3^high^ cells in canine lymphocytes and CD4+ CD8+ double-positive T cells. Although this effect did not achieve statistical significance, it is partly similar to data from humans as the levels of circulating Tregs are elevated in patients infected with *N. americanus*, a human hookworm closely related to *A. caninum* ^50^. In addition, we found AcES and TcES to cause marked upregulation of Helios within Foxp3^high^ lymphocytes. Helios is a member of the Ikaros transcription factor family that was initially thought to discriminate naturally occurring from peripherally induced Tregs^51^. Its expression has been also demonstrated in canine PBMCs and, based on the literature, the authors suggested that Helios might be a suitable marker for thymic-derived Tregs in dogs^48^. However, subsequent studies revised the hypothesis of Thornton *et al*.^51^ by showing that Helios expression can be induced in Foxp3+ T cells^52^ and by demonstrating Helios+ and Helios- cells within natural occurring Tregs^53^. Functionally, Helios has been linked to T cell activation and proliferation^54^, and its expression is necessary for a stable inhibitory Treg activity^55^. Although functional studies are needed to confirm the impact of Helios in dogs, its upregulation by canine Foxp3^high^ lymphocytes after treatment with ES products suggests *A. caninum* and *T. canis* larvae to activate canine Tregs and to improve their regulatory activity.

The mechanism by which parasites can raise the Treg frequency may encompass direct and indirect effects. For instance, Treg induction may be facilitated by a parasite-related homeostatic environment, which includes the shift to a pro-regulatory cytokine milieu^56^. Moreover, parasites can support Treg generation by modulating DCs, for example by preventing their maturation, which may then guide naïve T cells to differentiate into a regulatory subset. This has been illustrated for *H. polygyrus* or *Echinococcus granulosus* in which treatment of DCs with respective ES antigens elevate their Treg-inducing capacities^57, 58^. The results presented here show both AcES and TcES to impair upregulation of MHC-II and CD80 after TLR4 stimulation, thus implicating *A. caninum* and *T. canis* to be capable of reducing DC maturation in dogs. However, further studies should verify whether DCs pulsed with AcES or TcES are indeed more competent in modulating canine Tregs. Interestingly, AcES seems to be more potent in reducing the MHC-II expression of moDCs than TcES. Despite the fact that this could implicate different modes of action by which AcES and TcES influence DCs, the effect may further be related to quantitative differences in the fractions of ES antigens responsible for this finding. In addition to the interaction with DCs, parasites can directly drive Treg differentiation by secreting homologues that mimic the function of host molecules. For instance, this has been demonstrated for ES products of *H. polygyrus* that induce expression of Foxp3 via a TGF-β-like signalling pathway^16^. The identification of *Ac-daf-7* and *Ac-dbl-1* in larval secretions of *A. caninum*, both of which are TGF-β-like ligands with homology to proteins of *Caenorhabditis elegans*, may implicate AcES to be capable of influencing T cells independent from DCs^59, 60^. Therefore, conditions required for Treg induction and/or modulation by AcES and TcES in dogs should be further addressed in future studies to better define whether this is dependent on or independent of the presence of DCs.

Confirming the immunoregulatory potential of AcES and TcES in the definitive host on the cytokine level, we show ES antigens to enhance IL-10 secretion of canine lymphocytes. This is in accordance with previous studies on the cytokine pattern induced by larval secretions of *A. caninum* and *T. canis* in humans and/or mice showing them to be dominated by anti-inflammatory molecules including IL-10^17, 35, 36^. Similarly, PBMCs of dogs infected with *T. canis* during pregnancy are reported to show higher secretion of IL-10^39^. IL-10 is one of the most important anti-inflammatory cytokines as reviewed by Sabat *et al*.^61^ and its ablation induces severe failure in immune homeostasis causing inflammatory disorders such as colitis^62^. In the context of helminth infection, parasite-induced IL-10 secretion may enhance the homeostatic capacity of the host including elevated generation of Tregs^63^. In addition, IL-10 may also interfere with the maturation of antigen-presenting cells by preventing the transport of peptide-laden MHC-II molecules to the cell surface^64^. This could represent a possible mechanism by which AcES and TcES stop MHC-II upregulation by LPS-stimulated canine moDCs.

Three-colour flow cytometry revealed CD8+ T cells to be mainly responsible for ES-induced IL-10 expression. Although their marked increase in IL-10 suggests a regulatory function, it remains speculative whether they represent CD8+ Tregs. Being present in much lower frequencies than their CD4+ counterpart, CD8+ Foxp3+ Tregs have been described in humans, mice and dogs^48, 65^. In the context of helminth infection, Cuéllar *et al*. showed murine splenic CD8+ cells, which are further characterised by the expression of Foxp3, CD25 and remarkable amounts of IL-10, to be responsible for suppressive effects of the recombinant metalloproteinase Ac-TMP-2 derived from adult *A. caninum* ^15^. This implicates an important role of IL-10 in murine splenic CD8+ Tregs, which has been substantiated by others who found IL-10 to be required for the suppressive capacity of this cell population^66^. Interestingly, in murine *H. polygyrus* infection, CD8+ Tregs isolated from the intestinal lamina propria were capable of suppressing splenocyte proliferation in a contact-dependent manner unrelated to the presence of IL-10^67^. The results of the present study suggest that IL-10+ CD8+ T cells play a significant role in helminth-induced immune regulation by *T. canis* and *A. caninum* in dogs. Therefore, future studies should continue in characterising their phenotype and regulatory capacity, and thereby elucidating the role of CD8+ T cells in canine helminth infection.

Interestingly, ES treatment prior to LPS stimulation caused mildly increased IL-17 expression in IL-10+ lymphocytes. However, it remains unclear whether these IL-10+ IL-17+ double-positive cells represent IL-10-producing Th17 cells or if this finding just reflects IL-17 expression by other lymphocytes. Considering the upregulation of IL-17 in circulating Tregs of *N. americanus*-infected human patients^50^ and in the peripheral blood of *T. canis*-infected mice^38^, subsequent studies might perform co-localisation of IL-10, IL-17 and other Th-specific molecules to further elucidate the phenotype of canine ES-induced IL-10+ IL-17+ cells.

Besides the analysis of antigen-specific effects, proliferation assays have been used to investigate whether parasites are capable of reducing polyclonal T cell activation. While this can be indirectly achieved by parasite-induced Tregs^15, 16^, several studies provide evidence of worm-related factors by which they can directly interfere with lymphocyte proliferation in response to mitogens as demonstrated for *Ascaris lumbricoides*, *H. polygyrus*, *N. americanus* and *Trichuris trichiura* ^50, 68, 69^. Similar effects have also been reported for *A. caninum* when adult worm extracts or living infective larvae were added to human mitogen-stimulated PBMCs^17^. Interestingly, the anti-proliferative effect was elevated in those patients infected with *N. americanus* ^17^. In the present study, we demonstrate that larval secretions of *A. caninum* and *T. canis* inhibit the proliferation of canine PHA-stimulated T cells. With respect to the mechanism responsible for this effect, the anti-proliferative potential of adult worm secretions of *N. americanus* on human PBMCs is directly dependent on the presence of CD4+ CD25+ Foxp3+ Tregs^50^. Moreover, this effect may be further attributed to CD8+ Tregs as they are shown to outrun the suppressive capacity of CD4+ Tregs on lymphocyte proliferation^15, 67^. To better define the circumstances essential for the inhibition of polyclonal immune cell activation during canine helminth infection, additional studies should investigate the contribution of soluble and cellular factors.

In summary, we demonstrated several effects by which *A. caninum* and *T. canis* can interfere with the canine immune system. These include the induction and/or modulation of Foxp3^high^ T cells, an increase in CD8+ IL-10+ T cells, inhibition of polyclonal T cell proliferation and the prevention of DC maturation. These effects are mediated by secretions of infective larvae, suggesting that the anti-inflammatory capacities already evolve early in the parasitic life-cycles, i.e. in the pre-adult stages. Remarkably, the present study further shows many of the immunomodulatory effects to be mediated by both nematodes. Considering the differences between *A. caninum* and *T. canis*, which are not only obvious on a phylogenetic level but also illustrated by sodium dodecyl sulphate polyacrylamide gel electrophoresis (SDS-PAGE) of ES antigens or proteomic studies^29, 31^, this may raise the question whether the findings presented here are related to common molecules shared by both organisms or attributed to distinct mechanisms that differ between both species. Therefore, future studies may focus on distinct ES fractions or single molecules to further increase the understanding of parasite-related immune regulation.

## Material and Methods

### Ethics statement

All animal experiments were performed in accordance to the German Animal Welfare Act as well as national and international guidelines for animal welfare. The animal experiments were approved by the Institutional Animal Care and Use Committee (IACUC) of the Lower Saxony State Veterinary Office for Consumer Protection and Food Safety (Niedersächsisches Landesamt für Verbraucherschutz und Lebensmittelsicherheit, Oldenburg, Germany; registration numbers: 33.19-42502-05-01A038, 33.19-42502-05-15A587, 33.19-42502-05-16A024).

### Animals

For generating nematode larvae, dogs were experimentally infected with *T. canis* or *A. caninum*, respectively, at the Institute for Parasitology (University of Veterinary Medicine Hannover). For isolating PBMCs, peripheral blood was obtained from dogs of various breeds, which were presented to the Unit of Reproductive Medicine (University of Veterinary Medicine Hannover) due to reasons unrelated to the present study and samples were only collected after the owner’s agreement. In addition, peripheral blood was also obtained from Beagle dogs permanently housed in the Unit of Reproductive Medicine for reasons unrelated to the present study. All dogs were free from apparent disorders according to the clinical examination. Owners’ dogs were routinely treated with anthelminthic drugs before blood sample collection. In the case of Beagles, absence of intestinal parasites was checked by parasitological examination of faecal samples.

### Preparation of parasitic antigens

To obtain *T. canis* and *A. caninum* L3 for ES culture, eggs were obtained from faecal samples of experimentally infected dogs, these then being processed via the routine sedimentation-flotation technique. *T. canis* eggs were washed and embryonated in tap water at 25 °C for about 4 weeks. Larval hatching was carried out *in vitro* as described elsewhere^70^. For *A. caninum*, obtained eggs were incubated in a coproculture for about one week until development of L3. Larvae were exsheathed by incubation with sodium hypochlorite for 15 min at 37 °C.

*A. caninum* and *T. canis* L3 were washed approximately 10 times in sterile phosphate-buffered saline (PBS, pH 7.4) containing antibiotics (100 U/mL penicillin, 100 µg/mL streptomycin) followed by cultivation under standard conditions in RPMI-1640 supplemented with 1% glucose and antibiotics as follows: 100 U/mL penicillin, 100 µg/mL streptomycin, 50 µg/mL gentamycin, 0.5 µg/mL amphotericin B. Parasites were cultured for up to 5 weeks and culture dishes were monitored daily for larval viability (at least 80% viable larvae to obtain ES products) and absence of bacterial contamination. Tissue culture supernatants were collected weekly and concentrated using a centrifugal filtration unit with a 3 kDa cut-off (Vivaspin® Centrifugal Concentrators, Sartorius AG, Göttingen, Germany) according to the manufacturer’s recommendations. Extracts were then filtered through a low-binding disposable 0.22 µm filter and stored at -80 °C for up to 6 months until use. Protein concentrations of ES extracts were estimated by measuring the absorbance at 280 nm with a NanoDrop ND-1000 Spectrophotometer and results were calculated using logistic regression analysis based on a bovine gamma globulin standard curve (see Supplementary Method S1). SDS-PAGE showed no major differences between batches of ES extracts. ES preparations were further found to have a mean endotoxin content of 0.52 EU/mL as determined by using the Pierce™ LAL Chromogenic Endotoxin Quantitation Kit (Thermo Fischer Scientific, Waltham, Massachusetts, USA).

### PBMC isolation

For PBMC isolation, 20 mL of heparinised canine peripheral blood were diluted 1:2 in PBS followed by density gradient centrifugation (Histopaque®-1077, Sigma Aldrich, Taufkirchen, Germany) at 700 × *g* for 30 min. Mononuclear cells were collected and washed 3 times in PBS containing 0.02% 1 M ethylenediaminetetraacetic acid (EDTA). Isotone erythrolysis was applied to eliminate contaminating erythrocytes if appropriate. Cells were then resuspended in RPMI-1640 supplemented with 10% inactivated foetal calf serum (FCS) and antibiotics (100 U/mL penicillin, 100 µg/mL streptomycin).

### Cell culture

To analyse whether ES antigens are able to induce Tregs, PBMCs were cultured in 96 well microplates at 0.5 × 10^6^ cells/well and stimulated with ES antigens at 15 and 150 µg/mL, respectively, or medium under standard culture conditions. ConA, which is known to induce canine Tregs *in vitro* ^48^, was further used at 5 µg/mL as control. After 72 hours, cells were harvested and analysed by flow cytometry regarding their expression of Foxp3, CD4 and CD8, Foxp3 and Helios, as well as Foxp3 and CD25.

For investigating the potential of larval ES antigens to induce IL-10 production, PBMCs were cultured in 96 well microplates at 0.5 × 10^6^ cells/well and pulsed with ES products at 15 and 150 µg/mL, respectively, following stimulation with LPS at 1 µg/mL (LPS-B5 Ultrapure, InvivoGen, Toulouse, France) or medium after 3 hours. ConA, LPS and medium were used as controls. After 24 hours, culture supernatants were collected and stored at -80 °C until further use.

To investigate IL-10-expressing cells on the single cell level, intracellular cytokine staining was used. For this purpose, PBMCs cultured in 96 well microplates at 0.5 × 10^6^ cells/well were pulsed with AcES or TcES at 15 and 150 µg/mL, respectively. After 3 hours, cells were supplemented with 1 µg/mL LPS or medium and cultured for a further 18 hours. Brefeldin A was added during the final 12 hours at 5 µg/mL. Finally, PBMCs were stained for IL-10, CD4 and CD8, IL-10 and Foxp3, as well as IL-10 and IL-17, respectively.

To further analyse the potential of ES products to suppress mitogen-induced lymphocyte proliferation, PBMCs labelled with CFSE were pulsed with AcES or TcES at 15 and 150 µg/mL, respectively, for 3 hours following stimulation with PHA-L at 2.5 µg/mL in 96 well microplates at 0.5 × 10^6^ cells/well. BSA at 15 and 150 µg/mL, respectively, was used as additional controls. After 4 days, cells were stained for CD4 and CD8 and analysed by flow cytometry.

### MoDC maturation

To evaluate whether ES antigens are capable of modulating TLR4-mediated maturation of antigen presenting cells, moDCs were generated as previously described with few modifications^71^. Briefly, PBMCs were cultured at 2 × 10^6^ cells/mL in medium under standard culture conditions. After 24 hours, non-adherent cells were removed and adherent monocytes were cultured in the presence of 20 ng/mL canine recombinant IL-4 and 10 ng/mL canine recombinant GM-CSF for an additional 6 days. During this period, fully supplemented medium was changed after 3 days. Subsequently, moDCs were harvested by collecting non-adherent cells and pulsed with 150 µg/mL AcES or TcES for 24 hours in 96 well microplates (at least 20,000 cells/well). MoDCs were then stimulated with LPS at 1 µg/mL or medium for a further 24 hours. LPS was used as control. MoDCs were subsequently analysed regarding the expression of MHC-II and CD80 by flow cytometry.

### Flow cytometry

The following antibodies were used for surface staining: Rat anti-canine CD4 (clone YKIX302.9, Bio-Rad AbD Serotec, Puchheim, Germany) labelled with Lightning-Link® Rapid Atto633 (Innova Biosciences, Babraham, UK), rat anti- canine CD4:FITC (clone YKIX302.9, eBiosciences, Frankfurt am Main, Germany), rat anti-canine CD8:RPE and CD8:AF647 (clone YCATE55.9, Bio-Rad AbD Serotec), mouse anti-canine CD11c (clone CA11.6A1, obtained from Peter F. Moore, UC Davis, CA, US), mouse anti-canine CD25:PE (clone P4A10, eBiosciences), rat anti-canine MHC-II:FITC (clone YKIX334.2, Bio-Rad AbD Serotec) and hamster anti-mouse CD80:AF647 (clone 16-10A1, BioLegend, London, UK).

Antibodies for detecting intracellular antigens included polyclonal goat anti-canine IL-10 (AF735, R&D Systems, Wiesbaden, Germany) labelled with Lightning-Link® Rapid R-PE (Innova Biosciences), polyclonal goat anti-human IL-17 (AF-317-NA, R&D Systems), which has been recently shown to cross-react with canine IL-17^72^, labelled with Lightning-Link® Rapid FITC (Innova Biosciences), rat anti-mouse/rat Foxp3:eFluor660 (clone FJK-16s, eBiosciences) and hamster anti-mouse/human Helios:AF488 (clone 22F6, BioLegend).

For cell surface staining, cells were washed twice in PBS containing 1% BSA and 0.01% sodium azide (PBS-BSA) followed by incubation with primary antibodies on ice for 45 min. For staining of moDCs, PBS supplemented with 3% FCS, 1 mM EDTA and 0.01% sodium azide was used instead of PBS-BSA and Fc receptors were blocked by pre-incubation in 10% heat-inactivated dog serum for 15 min before adding primary antibodies. For staining canine CD11c, after washing steps, R-PE-labelled F(ab’)_2_-fragments of goat anti-mouse IgG (Dianova, Hamburg, Germany) were applied for 30 min on ice. Cells were then washed twice in PBS-BSA and either resuspended in BD FACSFlow™ Sheath Fluid (Becton Dickinson, Heidelberg, Germany) or further used for intracellular staining procedures.

For detecting intracellular antigens, cells were fixed and permeabilised using the Foxp3 / Transcription Factor Staining Buffer Set (eBiosciences) according to the manufacturer’s instructions. Cells were pre-treated with heat-inactivated mouse, rat and dog serum (each 15%) to block Fc receptors followed by incubation with antibodies for 45 min at ambient temperature. Cells were then washed and resuspended in FACSFlow™ Sheath Fluid.

Samples were analysed with a FACSCalibur^TM^ flow cytometer (Becton Dickinson) within 2 hours by collecting at least 60,000 events if available. Isotype-matched control antibodies were used as negative controls in equal concentrations. Data were analysed with FCS Express (De Novo Software) and FlowJo (Tree Star). Foxp3-stained lymphocytes were differentiated into a Foxp3+ and Foxp3^high^ population according to isotype controls (0.5% and 0.1%, respectively). CD25+ and CD25++ gates were set based on the 5.0% and 2.0% region of medium control lymphocyte cultures, respectively, which is in accordance to a previous investigation of canine Tregs^48^. IL-10+ lymphocytes were defined based on isotype controls (0.5% gate). For CD4, CD8 and IL-17 staining, positive cells were identified based on morphology as they were obviously detectable in flow cytometry plots.

### IL-10 ELISA

The amount of IL-10 in culture supernatants of stimulated PBMCs was determined using sandwich ELISA (Canine IL-10 Quantikine® ELISA kit, R&D Systems) according to the manufacturer’s instructions. Absorbance was measured at 450 nm and IL-10 concentrations were calculated using the 4-parameter logistic nonlinear regression model.

### Statistical analysis

Statistical analyses were performed using R version 3.3.0 (https://www.R-project.org/). Initially, data were checked for normal distribution by means of the Shapiro-Wilk test. Accordingly, the two-tailed Wilcoxon signed-rank test was used to evaluate differences in the amounts of IL-10 in supernatants of PBMC cultures without LPS stimulation (n = 6 dogs) and in expression of CD80 by moDCs (n = 6 dogs). The two-tailed paired t-test was performed to calculated differences in the percentage of Foxp3+/++ cells in the Treg stimulation assay (n = 6 dogs), to analyse differences in intracellular IL-10 expression (n = 6 dogs), to check for changes in IL-10 in supernatants of LPS-stimulated PBMCs (n = 4 dogs) and to evaluate differences in the expression of MHC-II (n = 6 dogs). One-way ANOVA followed by the Tukey’s honest significant difference post-hoc test was applied to evaluate differences in CFSE MFI for the proliferation assay (n = 5 dogs). P-values ≤ 0.05 were considered as statistically significant.

## Electronic supplementary material

Supplementary Information
