# Supplementary material for: Zoonotic intestinal helminths interact with the canine immune system by modulating T cell responses and preventing dendritic cell maturation
Source: Sci Rep. 2017 Sep 4;7:10310. doi: 10.1038/s41598-017-10677-4 (PMC5583179; doi:10.1038/s41598-017-10677-4)
Supplement: Supplementary file 1 — Supplementary Information [file 41598_2017_10677_MOESM1_ESM.pdf]

Supplementary Method S1

Zoonotic intestinal helminths interact with the canine immune system by modulating T cell responses and preventing dendritic cell maturation

Johannes Junginger[1], Katharina Raue[2], Karola Wolf[3,4], Elisabeth Janecek[2], Veronika M. Stein[4,#a], Andrea Tipold[4], Anne-Rose Günzel-Apel[3,4], Christina Strube[2], Marion Hewicker-Trautwein[1,*]

[1]Department of Pathology, University of Veterinary Medicine, Bünteweg 17, D-30559 Hannover, Germany
[2]Institute for Parasitology, Center for Infection Medicine, University of Veterinary Medicine, Bünteweg 17, D-30559 Hannover, Germany
[3]Unit of Reproductive Medicine of Clinics, University of Veterinary Medicine, Bünteweg 15, D-30559 Hannover, Germany
[4]Small Animal Clinic, University of Veterinary Medicine, Bünteweg 9, D-30559 Hannover, Germany
[#a]Current Address: Vetsuisse Faculty, University of Bern, Länggassstrasse 128, CH-3012 Bern, Switzerland

Determination of protein concentration in parasitic antigen preparations and comparison to other studies

Antigens were extracted from culture dishes containing high numbers of larvae (ranging from 50,000 to 500,000), consequently resulting in varying protein content of culture supernatants. All experiments described in the present study were performed using purified excretory-secretory (ES) extracts concentrated over Vivaspin columns. Protein concentrations of ES extracts were estimated by measuring the absorbance at 280 nm with a NanoDrop ND-1000 spectrophotometer and results were calculated using logistic regression analysis based on a bovine gamma globulin standard curve. This method of protein determination was chosen for the following reasons: (1) to minimise the handling time of ES extracts to avoid inactivation of the proteins we did not perform buffer exchange replacing the RPMI-1640 medium by phosphate buffered saline (PBS); (2) we found the commonly used protein determination kits to provide inconsistent results for the protein concentration in RPMI-1640-based extracts (without buffer exchange), which is most likely related to other medium ingredients present in the respective samples.

For the experiments of the present study, ES antigens were used at 15 or 150 µg/mL as determined by absorption at 280 nm. The upper protein concentration seems to be very high in comparison to other studies, mostly using ES antigens at approximately 10 µg/mL. However, the protein concentration is highly dependent on the standard reagent. For example, replacing the globulin standard by bovine serum albumin (BSA) resulted in protein concentrations twice as high as the globulin-based values.

Additional variations in protein concentration between this and other studies can, at least to some extent, be explained by the nature of the detection method itself. Results of the absorbance at 280 nm mainly depend on the proportion of tryptophan within the measured protein mix, which may differ between various samples (i.e., ES batches and standard controls). Contrary, the Bradford assay (being commonly used in other studies) allows a more comprehensive measurement of protein concentration. Although we were not able to re-evaluate all of the ES batches used in the present study, we performed several comparative tests between NanoDrop and Bradford assay (commercial Bradford assay kit that is compatible with several medium ingredients) using fresh batches of ES antigen. These experiments showed protein concentrations to be considerably lower when determined by the Bradford assay.

The comparison between Bradford and NanoDrop demonstrates that protein concentrations used in the present study are most likely, at least to some extent, comparable to that of other studies. In addition, this illustrates the importance of standardisation for determination of protein concentrations for such studies.
